# Patient-Level Determinants Outweigh Pharmacotherapy Choice in Smoking Cessation: Evidence from a Real-World Study in Turkey

**DOI:** 10.3390/healthcare14183090

**Published:** 2026-09-19

**Authors:** Hasan Hüseyin Mutlu, Feyzanur Erdem, Hacer Hicran Mutlu

**Affiliations:** 1 Department of Family Medicine, Faculty of Medicine, University of Health Sciences, İstanbul Sancaktepe Şehit Prof. Dr. İlhan Varank Health Practice and Research Center, İstanbul 34785, Turkey; hasanhuseyin.mutlu@sbu.edu.tr; 2 Department of Family Medicine, Prof. Dr. Cemil Taşcıoğlu City Hospital, Istanbul 34384, Turkey; 3 Department of Family Medicine, Faculty of Medicine, Istanbul Medeniyet University, Istanbul 34722, Turkey; hicran.mutlu@medeniyet.edu.tr

**Keywords:** smoking cessation, nicotine replacement therapy, pharmacotherapy, primary care, cytisine, bupropion

## Abstract

**Highlights:**

**What are the main findings?**

- In a real-world Turkish primary care cohort of 487 smokers, patient-level factors—education level, prior quit attempts, and duration of prior abstinence—independently predicted 3-month smoking cessation success, and bupropion plus NRT combination therapy was associated with significantly higher odds of cessation than cytisine.
- The overall 3-month cessation rate was 32.2%, with the highest (non-significant) rate observed for bupropion plus nicotine replacement therapy (47.4%).

**What are the implications of the main findings?**

- In resource-constrained settings where first-line agents such as varenicline are unavailable, individualized, behaviorally informed cessation strategies may matter more than the specific pharmacotherapy prescribed.
- These findings support prioritizing patient-centered counseling and relapse-prevention support alongside pharmacotherapy in primary care smoking cessation programs.

**Abstract:**

**Background/Objectives:** The relative contribution of pharmacotherapy versus patient-level factors to smoking cessation success remains unclear, particularly in real-world settings where access to first-line treatments such as varenicline is limited. This study aimed to compare the effectiveness of commonly used pharmacotherapies and to identify independent predictors of cessation in a primary care setting in Turkey. **Methods:** In this prospective observational study, 487 adult smokers attending a smoking cessation clinic in Istanbul, Turkey, were included. Participants received cytisine (*n* = 243), bupropion (*n* = 130), nicotine replacement therapy (NRT) (*n* = 8), bupropion plus NRT (*n* = 38), or a non-pharmacological intervention consisting of structured behavioral counseling without pharmacotherapy (*n* = 68), based on clinical judgment and patient preference. Baseline sociodemographic characteristics, smoking history, and nicotine dependence (Fagerström Test for Nicotine Dependence, FTND) were recorded. Smoking cessation at 3 months was assessed via telephone follow-up. Multivariable logistic regression was used to identify independent predictors of cessation. **Results:** The overall 3-month cessation rate was 32.2% (138/429). Bupropion plus NRT combination therapy was associated with significantly higher odds of cessation than cytisine (aOR = 2.36, 95% CI: 1.10–5.08, *p* = 0.028), while bupropion, NRT, and non-pharmacological treatment did not differ significantly from cytisine; given the very small NRT subgroup (*n* = 8), this estimate should be interpreted cautiously. Among patient-level factors, higher educational level (aOR = 1.38, 95% CI: 1.02–1.85, *p* = 0.035), a prior quit attempt (aOR = 2.14, 95% CI: 1.14–4.00, *p* = 0.018), and a longer duration of prior abstinence (aOR = 1.01 per month, 95% CI: 1.00–1.02, *p* = 0.034) were independently associated with higher odds of cessation. Age, sex, employment status, number of previous quit attempts, and FTND score were not significant predictors. These findings should be interpreted with caution given the non-randomized design, short follow-up, and self-reported, biochemically unverified outcome ascertainment described below. **Conclusions:** In this real-world cohort from Turkey, bupropion plus NRT combination therapy, along with several patient-level factors, was independently associated with 3-month smoking cessation. These findings suggest a potential benefit of combination pharmacotherapy that warrants confirmation in larger, adequately powered studies, and highlight the continued importance of individualized, behaviorally informed cessation strategies, particularly in settings with constrained access to first-line treatments such as varenicline.

## 1. Introduction

Tobacco use continues to be one of the leading preventable causes of disease and death worldwide. Although it varies by country, an estimated 1.3 billion people use tobacco products [1]. “Tobacco use” encompasses a heterogeneous set of products—including combustible cigarettes, smokeless tobacco, pipes, cigars, waterpipes (hookah), and e-cigarettes—that differ substantially in composition, use patterns, and health risk profile; a substantial proportion of tobacco users worldwide, and in Turkey specifically, use products other than manufactured cigarettes. The present study is concerned specifically with cessation of combustible cigarette smoking, and the terms “smoking” and “cigarette smoking” are used throughout to refer to this product category unless otherwise specified. In 2012, the prevalence of daily cigarette smoking among people aged 15 and older was estimated at 31.1% for men and 6.2% for women [2]. The harmful effects of cigarette smoking on health encompass a wide range of chronic diseases, including cardiovascular and respiratory system diseases, as well as various types of cancer [3,4]. Beyond its direct physiological effects, cigarette smoking is known to be associated with exacerbating mental health disorders, contributing to an increased prevalence of anxiety and depressive disorders among smokers [5,6].

Conversely, smoking cessation confers substantial and immediate benefits for both physical and mental health. Evidence demonstrates that quitting significantly reduces the risk of stroke, myocardial infarction, and smoking-related malignancies [4,7]. Furthermore, an emerging body of research indicates that individuals who successfully cease tobacco use often experience marked improvements in mood and reductions in anxiety symptoms, underscoring a complex but profoundly beneficial interplay between smoking cessation and psychological well-being [5,6].

Quitting smoking has two important effects on public health. First, it reduces the burden of smoking-related diseases on the healthcare system, both economically and clinically; second, it significantly reduces exposure to second-hand smoke, which is a primary cause of preventable disease in non-smokers [8]. Educational interventions play a critical role in this process, as raising public awareness about the risks of tobacco use has been associated with greater motivation to quit smoking [9]. Evidence-based pharmacological options have been developed for this purpose. Bupropion reduces withdrawal symptoms by acting on dopamine and norepinephrine [10]. Cytisine, a plant-based alkaloid, has a similar effect by targeting nicotine receptors [11]. Nicotine replacement therapy (NRT) provides the body with nicotine in a controlled manner while limiting exposure to the harmful toxins present in cigarette smoke [12]. Varenicline, a partial agonist, alleviates withdrawal symptoms while also blocking nicotine’s rewarding effects [13,14,15]. Comparative studies have shown that varenicline alone achieves higher smoking cessation rates than bupropion or NRT [16,17]. However, combination pharmacotherapy has the potential to further increase smoking cessation success; for example, combining varenicline with bupropion increased prolonged abstinence compared to varenicline alone in a randomized trial [18]. Meta-analysis findings confirm that combination therapies—such as varenicline plus bupropion, or bupropion plus NRT—demonstrate superior short-term efficacy compared to monotherapies, although this advantage may not always persist at longer-term follow-up [19,20].

Despite these advances, smoking cessation remains a complex endeavor influenced by individual factors—including genetic predisposition, psychiatric comorbidity, socioeconomic status, and behavioral patterns—as well as by variations in clinical practice and access to care. Understanding the relative effectiveness of available pharmacotherapies is essential for aligning treatment protocols with current clinical guidelines and optimizing patient outcomes. For instance, a large UK primary-care cohort study found that 28.8% of patients prescribed varenicline were abstinent at 2 years, compared with 24.3% of those prescribed NRT (adjusted OR = 1.26, 95% CI: 1.23–1.29) [16]. Such disparities highlight the necessity for continued research to identify predictors of success and to tailor interventions to individual patient profiles [13].

Access to pharmacotherapy also varies considerably across healthcare systems. In Turkey, varenicline has been unavailable for several years, and cytisine has only recently been introduced into clinical practice as a low-cost alternative. This situation is not unique to Turkey: the global recall and discontinuation of varenicline production because of nitrosamine contamination in 2021 left the drug largely or fully unavailable across much of the United Kingdom, the European Union, Japan, and large parts of North and South America, and cytisine has increasingly been proposed or adopted as an alternative pharmacotherapy in several of these settings [21]. Turkey’s experience therefore reflects a broader international shift in smoking-cessation pharmacotherapy access rather than a locally unique scenario; nonetheless, the specific combination of complete varenicline unavailability and recent, subsidized cytisine introduction provides a useful real-world setting in which to examine whether cessation success is primarily driven by pharmacotherapy or by patient-level factors.

Therefore, this study aimed to compare the short-term effectiveness of cytisine, bupropion, NRT, and bupropion plus NRT combination therapy, and to identify independent predictors of smoking cessation success in a real-world primary care setting in Turkey.

## 2. Materials and Methods

This prospective observational cohort study was conducted at the Smoking Cessation Clinic of the Department of Family Medicine in a tertiary care hospital in Istanbul, Turkey. During the study period, varenicline was not available in Turkey, and cytisine had only recently been introduced as a low-cost alternative, shaping the therapeutic context of clinical practice.

A total of 487 adult smokers who presented to the clinic between 1 May and 1 August 2025 were consecutively enrolled. Eligible participants were aged 18 years or older, had been daily tobacco users for at least one year, and expressed an intention to quit smoking within one month. No exclusion criteria were applied regarding comorbid medical or psychiatric conditions in order to reflect routine clinical practice. All participants provided written informed consent prior to inclusion.

Treatment decisions were made jointly by the patient and the attending physician, taking into account clinical evaluation, contraindications, patient preferences, and drug availability. Participants received one of five treatment options: cytisine monotherapy, bupropion monotherapy, NRT, combination therapy with bupropion and NRT, or a non-pharmacological intervention consisting of structured behavioral counseling without pharmacotherapy. Cytisine was administered orally following the standard 25-day tapering regimen in accordance with national protocols: 1.5 mg every 2 h (up to 6 tablets/day) on days 1–3, gradually reduced in frequency, to 1–2 tablets daily by days 21–25. Sustained-release bupropion was initiated at 150 mg once daily for three days, followed by 150 mg twice daily for 7–12 weeks. NRT was provided in standard forms such as patches, gum, or lozenges at recommended doses. Combination therapy was prescribed based on clinical judgment. The non-pharmacological intervention consisted of structured behavioral counseling sessions delivered by clinic staff, without any pharmacological agent, for patients who declined or had contraindications to drug therapy. Counseling followed the clinic’s standard smoking-cessation protocol, delivered face-to-face by trained family medicine physicians and nursing staff, and typically comprised an initial 20–30 min session at enrollment followed by brief (10–15 min) reinforcement sessions at subsequent visits; content addressed motivation building, identification of triggers, coping strategies, and relapse prevention. However, the exact number, spacing, and fidelity of counseling sessions received by individual participants—in both the non-pharmacological and pharmacotherapy groups, all of whom also received brief counseling alongside medication—were not systematically logged in the clinical dataset, and medication adherence (e.g., pill counts, patch/gum usage diaries) was likewise not formally assessed; both are addressed as limitations below.

Baseline data were collected using a standardized Smoking Cessation Clinic Initial Assessment Form completed during face-to-face interviews. This included sociodemographic characteristics, smoking history, previous quit attempts, prior pharmacological treatments, and the longest duration of previous abstinence. Nicotine dependence was assessed using the validated Turkish version of the Fagerström Test for Nicotine Dependence (FTND) [22], originally developed by Heatherton et al. [23], with scores ranging from 0 to 10.

The primary exposure variable was the type of pharmacological treatment received. The primary outcome was continuous abstinence from smoking at three months, defined as self-reported complete abstinence without even a single puff during the follow-up period. Consistent with routine clinical practice at the study site, self-report was not corroborated by biochemical verification (e.g., exhaled carbon monoxide or salivary/urinary cotinine testing); the potential for misclassification bias from this approach is discussed in the Limitations. Outcomes were assessed via structured telephone interviews conducted three months after treatment initiation. Participants who could not be reached after three attempts (*n* = 58) were excluded from the primary (per-protocol/complete-case) analysis. To evaluate the influence of this exclusion, an intention-to-treat sensitivity analysis was additionally performed in which all participants lost to follow-up were conservatively classified as continued smokers (non-quitters); results of this sensitivity analysis are reported alongside the primary results.

Covariates included age, sex, marital status, educational level, employment status, smoking duration, age at smoking initiation, number of cigarettes smoked per day, previous quit attempts, duration of previous abstinence, and FTND score. These variables were selected based on prior literature and clinical relevance. Psychiatric comorbidity, current alcohol use, baseline motivation to quit (e.g., a validated readiness-to-change scale), and structured measures of social support were not captured on the standardized intake form and could therefore not be entered as covariates; this is acknowledged as a source of potential unmeasured confounding in the Limitations.

All statistical analyses were performed using SPSS version 25.0 (IBM Corp., Armonk, NY, USA) [24]. Continuous variables were assessed for normality using the Shapiro–Wilk test and are presented as mean ± standard deviation or median (minimum–maximum), as appropriate. Categorical variables are presented as frequencies and percentages. For comparisons among the five treatment groups, one-way analysis of variance (ANOVA) or the Kruskal–Wallis test was used for continuous variables, as appropriate, and the chi-square test or Fisher’s exact test was used for categorical variables; Student’s *t*-test or the Mann–Whitney U test was used for two-group comparisons where relevant.

To identify independent predictors of smoking cessation, multivariable logistic regression analysis was conducted using a forced-entry approach: treatment type and a pre-specified set of clinically relevant covariates were entered simultaneously, regardless of their univariate significance, rather than through stepwise selection. Results are presented as adjusted odds ratios (aORs) with 95% confidence intervals (CIs). Model fit was assessed using the Hosmer–Lemeshow test. A two-sided *p*-value < 0.05 was considered statistically significant. Treatment type was entered into the multivariable model as four indicator (dummy) variables (bupropion, NRT, bupropion plus NRT, and non-pharmacological treatment), with cytisine—the treatment received by the largest proportion of participants and the only medication provided free of charge at the study site—designated as the reference category. This allows each treatment-specific adjusted odds ratio to be interpreted as a pairwise comparison against cytisine, rather than as a linear trend across an arbitrarily ordered set of nominal categories. Model diagnostics included the Hosmer–Lemeshow goodness-of-fit test and assessment of multicollinearity using variance inflation factors (VIFs). Because an a priori power analysis was not performed, sample size adequacy for the multivariable model was evaluated post hoc using the events-per-variable (EPV) heuristic. Based on the final analytic sample (*n* = 429), 138 cessation events, and 12 model parameters (eight covariates, with treatment type represented by four indicator variables), the model yielded approximately 11.5 events per variable, which exceeds the conventional minimum of 10 events per variable recommended for stable logistic regression estimates.

To minimize bias, all consecutive eligible patients were included, and standardized data collection procedures were used. Although treatment allocation was nonrandomized, potential confounders were measured and adjusted for via multivariable logistic regression. Formal propensity-score adjustment or matching was considered as an alternative approach to confounding by indication; however, given the marked imbalance in group sizes (as few as *n* = 8 in the NRT arm) and the limited number of measured baseline covariates available for balancing, a propensity-score model could not be reliably estimated without risking severe overfitting and was therefore not pursued. Consequently, the multivariable-adjusted estimates presented here should be interpreted as adjusting for measured confounders only; residual confounding by indication (i.e., systematic differences in why patients or clinicians selected one treatment over another) cannot be excluded and is discussed further in the Limitations. Given the observational design and subgroup sample size limitations, no formal interaction analyses were performed beyond the intention-to-treat sensitivity analysis described above. The small NRT and combination-treatment groups resulted in wide confidence intervals and limited precision for treatment-specific estimates.

## 3. Results

**Figure 1:**
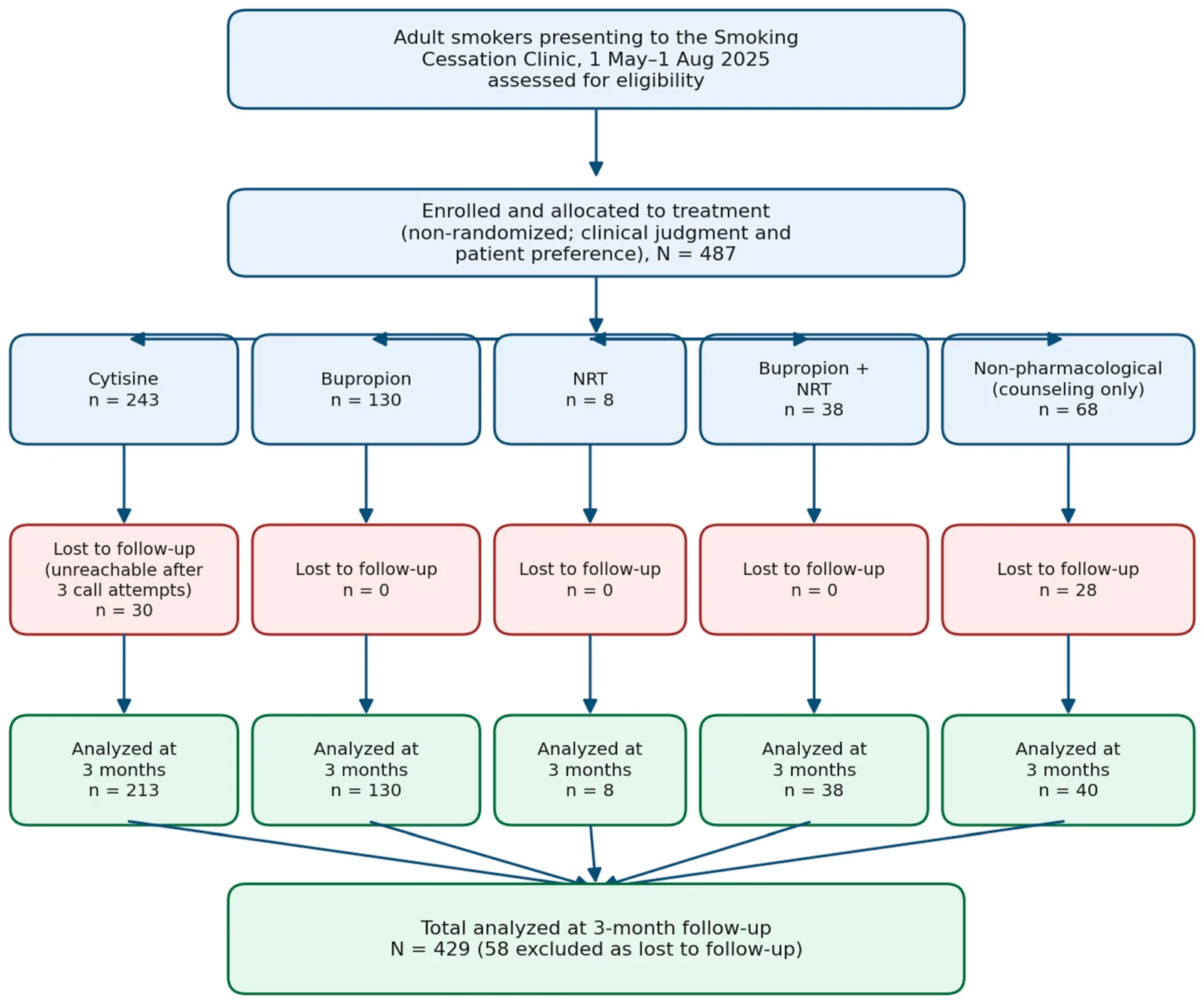
STROBE flow diagram of participant recruitment, non-randomized treatment allocation, loss to follow-up, and final analysis at the 3-month endpoint. Attrition was concentrated in the cytisine and non-pharmacological (behavioral counseling only) groups; the bupropion, NRT, and bupropion + NRT groups had negligible loss to follow-up.

A total of 487 participants were included in the study. At the three-month follow-up, 58 participants were lost to follow-up (unreachable by telephone after three attempts), and 429 individuals were included in the final analysis (Figure 1). Group-specific attrition was concentrated in the cytisine group (30 of 243 enrolled lost) and the non-pharmacological intervention group (28 of 68 enrolled lost), with negligible attrition in the bupropion, NRT, and bupropion plus NRT groups.

The median age of the overall cohort was 39 years (range: 18–74); age was approximately normally distributed in the cytisine and bupropion groups (presented as mean ± SD in Table 1) but not in the remaining groups, which are presented as median (range). Overall, 45% of participants were female (*n* = 219), with notable variation between groups; the proportion of women ranged from 25.0% in the NRT group (*n* = 2/8) to 65.8% in the bupropion plus NRT group (*n* = 25/38).

Most participants were married (59.1%, *n* = 288), with similar distributions across treatment groups. University graduates constituted 47% of the total sample (*n* = 229), with a higher proportion observed in the cytisine group (53.1%). Only a small proportion of participants were illiterate (1.2%, *n* = 6). Overall, 65.9% of participants were employed, although employment rates varied between groups, ranging from 44.7% in the combination therapy group to 75.3% in the cytisine group. Taken together, Table 1 shows that the five treatment groups differed appreciably in sex distribution, educational attainment, and employment status at baseline—differences expected under non-randomized, preference- and cost-driven treatment allocation rather than random chance—which reinforces the rationale for multivariable adjustment and for the confounding-by-indication discussion in the Limitations, since formal between-group significance testing was not performed for Table 1.

Regarding prior smoking cessation history, 9.7% of participants (*n* = 47) had previously used bupropion, 24.0% (*n* = 117) varenicline, and 12.9% (*n* = 63) NRT.

Smoking-related characteristics are summarized in Table 2. The median duration of smoking ranged from 17 years in the cytisine group to 22.5 years in the bupropion group. The mean age at smoking initiation was similar across groups (18.3 years, *p* = 0.99). Daily cigarette consumption ranged between 1.2 and 1.3 packs, with no significant difference between groups (*p* = 0.34).

The proportion of participants with previous quit attempts ranged from 67.6% to 100% across groups (*p* = 0.17). The median duration of previous abstinence ranged from 1 to 3.5 months (*p* = 0.14). Previous pharmacological treatment for smoking cessation was reported by 34.5% of participants (*n* = 168), with no significant difference between groups (*p* = 0.27). Mean FTND scores ranged from 6.05 to 7.38, indicating moderate-to-high nicotine dependence, without significant variation across treatment groups (*p* = 0.36).

**Figure 2:**
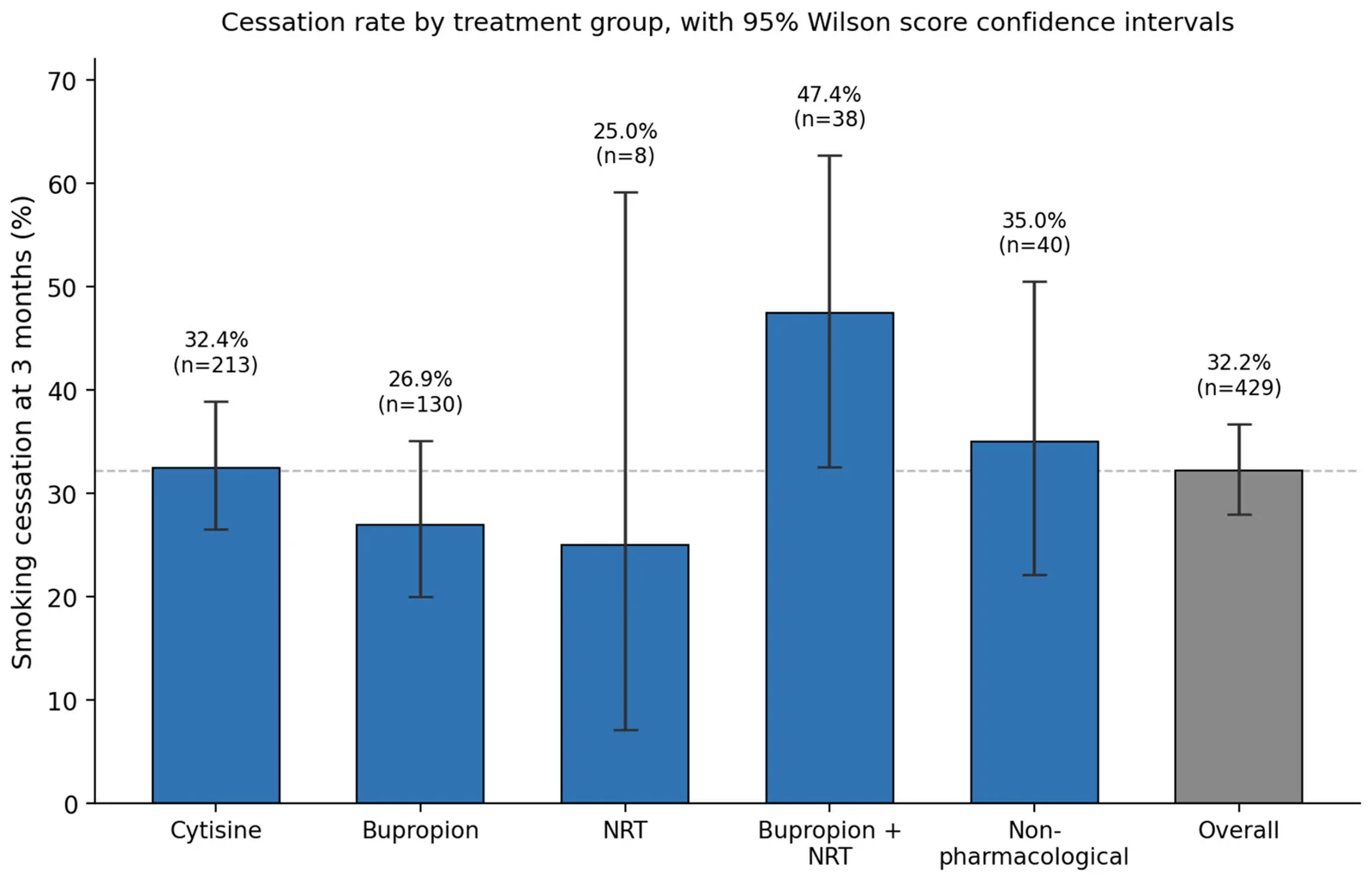
Smoking cessation rate at 3 months by treatment group, with 95% Wilson score confidence intervals. Error bars reflect the small size of the NRT (*n* = 8) and bupropion plus NRT (*n* = 38) arms; the dashed line marks the overall cessation rate (32.2%).

At the three-month follow-up, 32.2% of participants (*n* = 138/429; 95% CI: 27.9–36.7%) achieved smoking cessation. Absolute cessation rates with 95% Wilson score confidence intervals by group were cytisine 32.4% (69/213; 95% CI: 26.5–38.9%), bupropion 26.9% (35/130; 95% CI: 20.0–35.1%), NRT 25.0% (2/8; 95% CI: 7.1–59.1%), bupropion plus NRT 47.4% (18/38; 95% CI: 32.5–62.7%), and non-pharmacological (behavioral counseling only) 35.0% (14/40; 95% CI: 22.1–50.5%) (Figure 2). The highest point estimate was observed in the bupropion plus NRT group and the lowest in the NRT monotherapy group; however, the wide, overlapping confidence intervals—particularly for NRT and bupropion plus NRT, both derived from very small subgroups—indicate substantial imprecision, and differences between treatment groups were not statistically significant (*p* = 0.20). Given this imprecision, the apparent advantage of combination therapy should not be interpreted as evidence of superior effectiveness. In an intention-to-treat sensitivity analysis that conservatively classified all 58 participants lost to follow-up as continued smokers, the overall cessation rate was 28.3% (138/487; 95% CI: 24.5–32.5%); group-specific intention-to-treat rates were 28.4% for cytisine (69/243, reflecting its higher attrition), 20.6% for the non-pharmacological group (14/68, likewise affected by higher attrition), and unchanged for bupropion, NRT, and bupropion plus NRT (which had negligible loss to follow-up). The pattern of group differences was materially unchanged under this conservative assumption.

**Figure 3:**
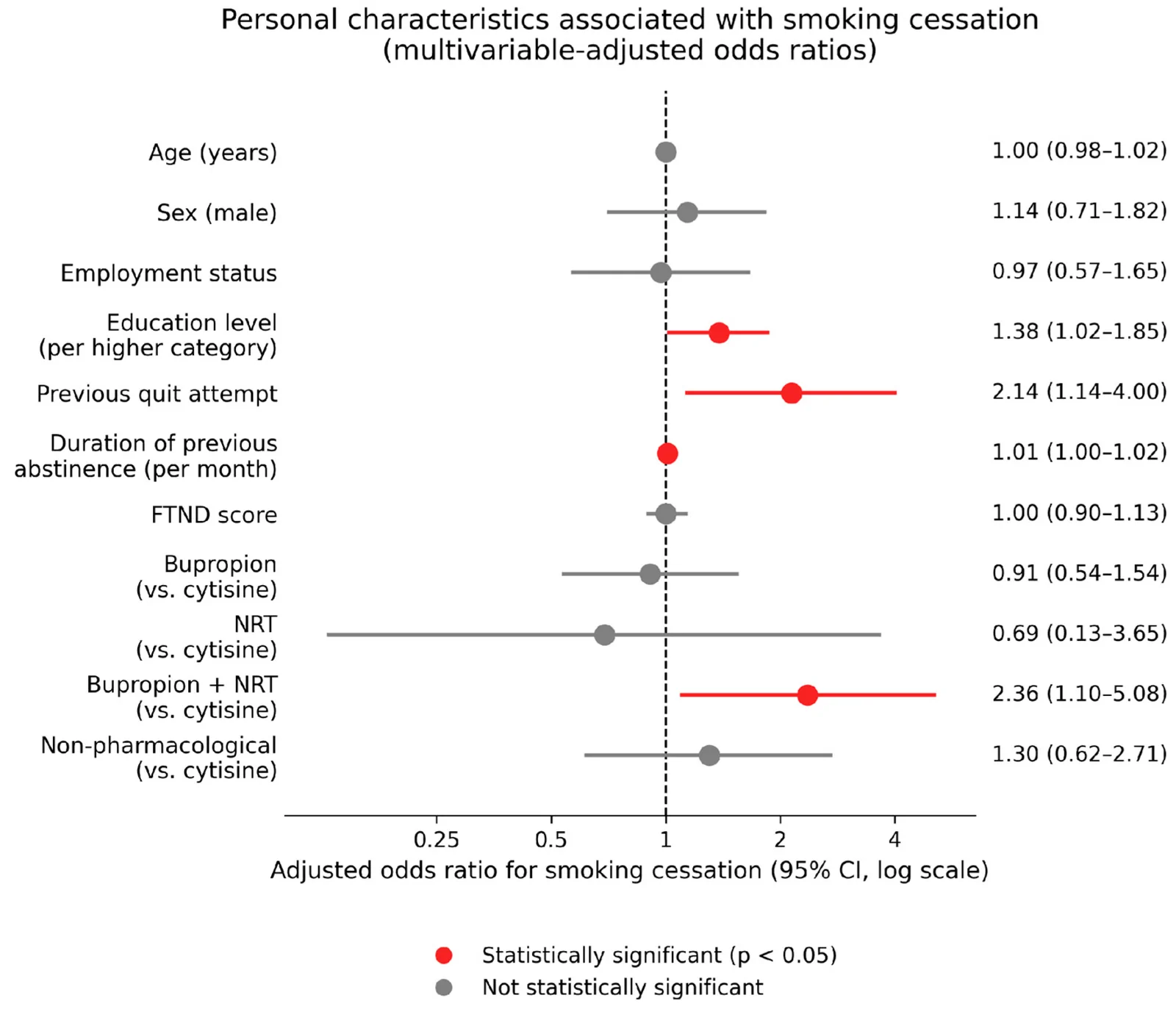
Personal characteristics and treatment type associated with smoking cessation: multivariable-adjusted odds ratios (95% CI) from the logistic regression model in Table 3, with treatment type entered as indicator variables (reference = cytisine). Points to the right of the reference line (aOR = 1) indicate higher odds of cessation; red markers denote statistically significant associations (*p* < 0.05). The NRT estimate (*n* = 8) has a wide confidence interval and should be interpreted cautiously.

Multivariable logistic regression (Table 3), with treatment type entered as indicator variables against cytisine as the reference category, showed that bupropion plus NRT combination therapy was associated with significantly higher odds of cessation compared with cytisine (aOR = 2.36, 95% CI: 1.10–5.08, *p* = 0.028). Neither bupropion monotherapy, NRT monotherapy, nor non-pharmacological treatment differed significantly from cytisine. Education level (aOR = 1.38, 95% CI: 1.02–1.85, *p* = 0.035), a previous quit attempt (aOR = 2.14, 95% CI: 1.14–4.00, *p* = 0.018), and longer duration of previous abstinence (aOR = 1.01 per month, 95% CI: 1.00–1.02, *p* = 0.034) were independently associated with higher odds of cessation (Figure 3). Age, sex, employment status, number of previous quit attempts, and FTND score were not significantly associated with the outcome. The model showed adequate goodness-of-fit (Hosmer–Lemeshow χ^2^ = 4.086, df = 8, *p* = 0.849), with no evidence of problematic multicollinearity (VIF 1.02–1.43). Given the small size of the NRT subgroup (*n* = 8), the corresponding estimate is imprecise and should be interpreted with caution.

The model demonstrated high specificity (96.5%) but low sensitivity (10.2%) in predicting cessation, with an overall classification accuracy of 68.8%.

## 4. Discussion

The observation that most participants in our study (74.5%) had a history of previous smoking cessation attempts is significant and highlights the complexity of smoking addiction and the challenges of quitting. This finding aligns with existing literature indicating that many smokers do not achieve long-term cessation after their initial attempts, often owing to a multifaceted interplay of behavioral, psychological, and physiological factors [25,26]. Bokoro et al. reported a similar rate (73.8%), which aligns closely with our findings [27]. Alasmari et al. reported that only 18.6% of participants in a study conducted in Saudi Arabia had at least one quit attempt, considerably lower than our result [28]. This discrepancy may be associated with sociocultural characteristics specific to the region where each study was conducted.

Smoking cessation rates were found to be 32.2% in our study. Qasem et al. reported a 13.6% quit rate in a general population online survey in Jordan, which is lower than our rate [29]. Uçar et al. reported that pharmacotherapies combined with behavioral support achieved smoking cessation rates of 32.5% (varenicline), 23% (bupropion), and 52.8% (NRT) at one year (overall 35%); the varenicline and NRT rates are broadly consistent with our findings, notably their participants were followed for one year, whereas ours were monitored for only three months [30]. In the QuLIT trial, similar to our findings, the cessation rate was 29.2% among participants who received both pharmacotherapy and counseling [31]. The variability in success rates across studies is often attributable to specifics of the intervention, including the intensity of support, duration of follow-up, and participant demographics.

A comprehensive analysis of prior studies indicates that cytisine enhances the likelihood of quitting smoking, with quit rates ranging from 8.4% to 12.1% depending on trial design and population [32,33]. In our study, the cessation rate was 32.4% among cytisine users, markedly higher than in previous studies. In our country, cytisine, NRT, and bupropion are currently the available options for smoking cessation treatment; among these, cytisine is the only medication patients can obtain free of charge, and therefore the majority of participants in our study used cytisine. The higher cessation rate observed for cytisine in our cohort relative to prior trials more plausibly reflects our short (3-month) follow-up window, self-reported and biochemically unverified outcome ascertainment, the motivated real-world clinic sample, concurrent behavioral counseling, and possible selection and attrition effects, rather than the predominance of cytisine use itself, which cannot mechanistically explain the group’s own success rate. A further caveat applies whenever cessation rates from studies with different follow-up durations are compared, including several of the comparisons below: cessation rates are well documented to decline substantially between early follow-up points (e.g., 1 or 3 months) and 12 months, with most relapse concentrated in the initial days and weeks after quitting, such that 12-month sustained abstinence can fall below the prolonged-abstinence rates seen even among untreated self-quitters [34]. Notably, relapse continues to occur even beyond the 12-month mark, at an estimated annual rate of approximately 10% among those who had already achieved one year of abstinence [35], underscoring that our own 3-month cessation rate should not be extrapolated to predict longer-term outcomes.

In studies conducted in Turkey, the smoking cessation rates associated with bupropion have been reported with varying results. In a centrally randomized cohort receiving free bupropion or varenicline through Turkey’s national smoking-cessation program, the continuous (7-day-verified) one-year abstinence rate among bupropion users was only 6.2% (10/161)—considerably lower than the 13.9% (34/244) observed with varenicline (*p* = 0.015)—while using a less stringent point-prevalence definition, the one-year bupropion abstinence rate in that same cohort was 18.6% [36]. Both figures are lower than the 26.9% bupropion cessation rate observed in our study; however, that comparison study assessed abstinence at one year rather than three months—a difference in follow-up duration that, per the relapse-curve evidence noted above, would generally be expected to yield a higher rate at three months than at one year, all else being equal, making the figures not directly comparable. Similarly, in another study evaluating three-month cessation rates, the rate among bupropion users was 35.6% [37], consistent with our findings. In contrast, a more recent retrospective Turkish study directly comparing bupropion, nicotine patch, and cytisine found a highly significant difference in cessation outcomes across pharmacotherapies, with cytisine (61.5%) substantially outperforming bupropion (16.3%) and nicotine patch (11.8%) (*p* < 0.001) [38]. This contrasts with our null finding for treatment type and may reflect differences in cytisine dosing, free-of-charge access driving greater motivation/adherence among cytisine users, or the larger sample size in that study providing greater power to detect between-group differences.

Although not statistically significant, the highest point-estimate cessation rate was observed in the bupropion plus NRT combination group (47.4%, 95% CI: 32.5–62.7%). This estimate is based on only 38 participants, and its wide confidence interval overlaps those of every other treatment group; it should therefore not be read as evidence that combination therapy outperformed the alternatives in this cohort, and we caution against overinterpreting this numerically highest but statistically inconclusive result. In a study conducted over a three-month period, cessation rates similar to ours were observed (40%) [39], whereas a study with a longer (6-month) follow-up duration reported a comparable combination-therapy abstinence rate of 24.2% [40]. Consistent with this pattern, a recent systematic review and meta-analysis found that the benefit of bupropion plus NRT over bupropion alone, while significant at end-of-treatment, became non-significant at long-term (≥6-month) follow-up (RR = 1.10, 95% CI: 0.90–1.34) [19], suggesting that the advantage of combination therapy may not be sustained over time.

When cessation rates for bupropion, NRT, bupropion plus NRT, and cytisine were compared, the difference among treatment groups was not statistically significant. Meta-analyses indicate that bupropion’s effectiveness may be comparable to that of NRT but does not surpass that of varenicline [41,42]. Recent systematic reviews highlight that cytisine has superior efficacy for smoking cessation compared with placebo. For instance, De Santi et al. reported that cytisine yielded a risk ratio (RR) of 2.25 compared with placebo, suggesting strong effectiveness for achieving cessation [43]. Additionally, Livingstone-Banks et al. noted that cytisine was among the treatments yielding higher cessation rates compared with placebo across 75 trials evaluated in their updated Cochrane review [44].

In longitudinal evaluations, both bupropion and NRT have demonstrated effectiveness; however, direct comparisons of cytisine with the combined use of these two treatments remain scarce. Dogar et al. reported that cytisine produced stronger cessation outcomes than placebo, particularly among high-risk groups (tuberculosis patients) [45]. Similarly, Stead et al. emphasized that integrating pharmacotherapy with behavioral support consistently improves cessation success regardless of the specific medication used, underscoring the importance of individualized treatment strategies [46]. A further meta-analysis revealed that varenicline combined with NRT yields higher quit rates than monotherapy, and that the combination of varenicline and bupropion outperformed both NRT and bupropion alone; varenicline monotherapy also showed greater efficacy than NRT or bupropion individually. In a subgroup analysis of that network meta-analysis pooling trials conducted in psychiatric populations broadly defined (predominantly studies in people with schizophrenia-spectrum and other severe mental illness, based on only a small number of included trials), the combination of bupropion and NRT was found to be more effective than placebo or bupropion alone [20]. This subgroup finding should not be extrapolated across all psychiatric diagnoses without qualification, since psychiatric disorders are managed very differently from one another and bupropion is not equally appropriate in all of them; in particular, bupropion lowers the seizure threshold [47] and, because of its noradrenergic and dopaminergic activity, carries a recognized risk of precipitating a manic or hypomanic switch in patients with bipolar disorder [48], so it is generally used with caution—if at all—in that population specifically, in contrast to its more established role in schizophrenia-spectrum disorders. In our study, no statistically significant difference was observed in efficacy between the pharmacological treatments; however, no participant used varenicline, owing to its unavailability in our country for the past 4 years. This finding may also be attributable to the relatively small and highly unequal group sizes—most notably the NRT (*n* = 8) and bupropion plus NRT (*n* = 38) arms—which yielded wide, overlapping confidence intervals around each group’s cessation rate and left the comparison underpowered (post hoc power = 64%) to detect anything short of a large true difference; it should not be read as evidence that these treatments are equally effective, only that this study could not distinguish between them. Unmeasured individual motivation, treatment adherence, and sample demographics may also have contributed. Additionally, the interplay between pharmacotherapy and patient response may differ widely among individuals.

Education level emerged as a significant independent predictor of cessation, with each higher educational category associated with higher odds of quitting (aOR = 1.38, 95% CI: 1.02–1.85, *p* = 0.035). This is consistent with a substantial body of prior work linking higher educational attainment to greater cessation success. Yang et al., for example, found that lower educational levels were associated with both higher smoking initiation and lower rates of cessation attempts, implicating education as a determinant of knowledge of and attitudes toward smoking risks and cessation methods [49]. Similarly, Abroug et al. reported that higher educational level was positively associated with smoking cessation [50], consistent with our finding. As with the other predictors identified here, this association should be interpreted with appropriate caution given the modest overall model fit (Nagelkerke R^2^ = 0.083) and the observational design.

The finding that individuals with a history of previous quit attempts had higher odds of success on the current attempt (aOR = 2.14, 95% CI: 1.14–4.00, *p* = 0.018) is consistent with a ‘learning model’ of cessation, whereby prior attempts—even unsuccessful ones—may build coping skills, self-efficacy, and familiarity with the quitting process that increase the likelihood of eventual success. This aligns with reports that repeated quit attempts are a normal and often necessary part of the pathway to long-term abstinence, rather than a marker of futility [51]. Supporting this perspective, Tran Luy et al. emphasized that temporary abstinence increases the probability of permanent cessation, highlighting that the number of previous quit attempts is a key determinant of long-term success and that the likelihood of quitting increases alongside the number of attempts [52]. Tran Luy et al. propose that individuals who have experienced recent failed attempts may have lowered expectations for future success, affecting their efforts to quit [52].

Notably, a validated Turkish adaptation of the Cessation Fatigue Scale administered in a comparable clinic-based population found no significant association between fatigue scores and nicotine dependence severity, and the scale did not reliably predict cessation success, although successful quitters did show lower initial fatigue scores than those who continued smoking [53]; this suggests that cessation fatigue may interact with quitting success in ways not fully captured by our study or by simple fatigue measures. Furthermore, a longer duration of prior abstinence was associated with a modest increase in the odds of achieving cessation during the current attempt (aOR = 1.01 per month, 95% CI: 1.00–1.02, *p* = 0.034). This suggests that individuals who have previously demonstrated a greater capacity for sustained abstinence—despite eventual relapse—retain coping skills and motivational resources that enhance the likelihood of success in subsequent quit attempts. While the broader literature indicates that the predictive effect of prior abstinence duration on current cessation outcomes is often statistically small or inconsistent, findings from Borland et al. provide empirical support for this positive association [54].

In conclusion, after adjustment for patient-level covariates, bupropion plus NRT combination therapy was associated with significantly higher odds of cessation than cytisine, although this estimate warrants confirmation given the modest sample size. Education level, previous quit attempts, and duration of prior abstinence were also significant predictors of cessation. These findings highlight both a potential benefit of combination pharmacotherapy and the importance of individualized, behaviorally supported approaches in smoking cessation programs.

### 4.1. Limitations

This study has several important limitations. First, its nonrandomized, observational design precludes causal inference regarding treatment effectiveness. Treatment selection was influenced by patient preference, clinician recommendation, and policy-driven access (e.g., free cytisine), introducing potential selection bias. For example, individuals opting for combination therapy may have been more motivated or had greater social support, factors not fully captured in our model.

Second, group sizes were highly imbalanced, particularly in the NRT (*n* = 8) and combination therapy (*n* = 38) arms, reducing statistical power and increasing the risk of a Type II error. The small size of some treatment subgroups limits the precision of treatment-specific estimates and increases the risk of failing to detect clinically meaningful differences between treatment groups. The NRT estimate (*n* = 8) in particular should be interpreted cautiously given its wide confidence interval. We also note that correcting the treatment-coding specification altered the estimated associations for education level and previous quit attempts, both of which reversed direction relative to the earlier, misspecified model; we believe this reflects the fact that the previous linear treatment term had absorbed variance properly attributable to treatment group.

Third, behavioral counseling was not standardized or quantified. Although all participants attended a cessation clinic, the frequency, duration, content, and quality of behavioral support were not recorded. Given that behavioral interventions significantly amplify pharmacotherapy efficacy [44], unmeasured variation in counseling intensity likely confounded the observed outcomes.

A related and important limitation is that smoking status was ascertained solely through a telephone self-report, without objective biochemical verification (e.g., exhaled carbon monoxide or salivary/urinary cotinine testing). This is a recognized source of misclassification bias in real-world smoking-cessation research: validation studies typically find that unverified self-report modestly overstates true abstinence, since some participants who have resumed smoking nonetheless report having quit, particularly when interviewed by staff connected to the clinic that treated them. This could have inflated the absolute cessation rates reported here (including the overall 32.2% rate and the group-specific rates presented with their 95% CIs), and, if the degree of over-reporting differed systematically across treatment arms or between more and less motivated participants, it could also have biased the comparisons between pharmacotherapies and the estimated predictors of cessation (Table 3) in either direction. We cannot determine the extent or direction of this bias from our data, and the true cessation rates may be somewhat lower than reported; this should be borne in mind when interpreting both the absolute rates and the between-group comparisons. Relatedly, medication adherence was not formally assessed; this too is a recognized source of misclassification bias in real-world smoking-cessation research and could have differentially affected treatment arms if adherence or the social desirability of reporting success varied by treatment type or by whether counseling alone (versus a prescribed medication) was received.

Because treatment allocation was non-randomized and shaped substantially by cost and availability (free cytisine versus out-of-pocket bupropion, NRT, or combination therapy), confounding by indication is a central concern. We adjusted for measured covariates in the multivariable model but did not perform formal propensity-score adjustment, given the small size of some treatment arms; important baseline characteristics that plausibly influence both treatment selection and cessation success—psychiatric comorbidity, alcohol use, baseline motivation or readiness to quit, and social support—were not systematically recorded and could not be included as covariates or balancing variables. Residual confounding from these unmeasured factors, rather than a true absence of pharmacotherapy effect, cannot be ruled out as an explanation for the null finding on treatment type.

Our primary analysis excluded the 58 participants (11.9%) lost to telephone follow-up rather than analyzing all enrolled participants on an intention-to-treat basis; attrition was concentrated in the cytisine and non-pharmacological groups, raising the possibility of differential loss to follow-up by treatment arm. A conservative intention-to-treat sensitivity analysis, treating all participants lost to follow-up as continued smokers, produced a materially similar pattern of results (overall cessation 28.3% vs. 32.2% in the complete-case analysis), which is reassuring but does not eliminate the possibility of bias if reasons for being unreachable were themselves related to smoking status.

Fourth, cessation was assessed at only three months, limiting insight into long-term abstinence. Relapse is common beyond this window, and early success does not always translate into sustained quitting.

Finally, varenicline has been unavailable in Turkey since 2021, restricting the range of pharmacotherapies studied and preventing comparison with what meta-analyses consistently identify as the most effective first-line agent [19,40].

### 4.2. Interpretation

Taken together, these findings suggest that, in this real-world setting, cessation outcomes were associated with both patient-level characteristics and, notably, the choice of combination pharmacotherapy. The lack of statistically significant differences across treatment groups likely reflects methodological constraints—particularly non-randomization and insufficient power—rather than true therapeutic equivalence. The logistic regression model accounted for only 8.3% of the variance in cessation status (Nagelkerke R^2^ = 0.083) and demonstrated low sensitivity (10.2%) alongside high specificity (96.5%). This limited predictive ability most likely reflects the omission of key variables not captured in our dataset—such as psychiatric comorbidity, alcohol use, baseline motivation, social support, and medication adherence—rather than a genuine absence of predictable structure in cessation behavior. Motivation and adherence warrant particular mention; in a recent Italian cohort of cytisine users, adherence strongly predicted success (75% vs. 25%, *p* = 0.011), yet baseline motivation scores did not differ between adherent and non-adherent patients, underscoring a complex interrelationship [55]. As we did not measure these factors, their contribution to our findings remains unknown and should be addressed in future investigations.

Within this constrained model, higher education, previous quit attempts, longer prior abstinence, and bupropion plus NRT emerged as independent predictors. Higher educational attainment was associated with greater odds of success (aOR = 1.38, 95% CI: 1.02–1.85), consistent with literature linking socioeconomic status to favorable health behaviors [50,51]; however, this association should be interpreted cautiously given the potential for unmeasured confounding.

A history of previous quit attempts also increased the odds of current success (aOR = 2.14, 95% CI: 1.14–4.00). Rather than indicating “cessation fatigue,” this finding supports a “learning model” in which prior attempts—even unsuccessful ones—foster coping skills, self-efficacy, and familiarity with the quitting process, thus representing a normal pathway to sustained abstinence [51,52]. As Tran Luy et al. noted, temporary abstinence increases the probability of permanent cessation, although the impact of prior attempts may vary depending on their recency and circumstances [52]. Similarly, a longer duration of prior abstinence was associated with a modest increase in the odds of current success (aOR = 1.01 per month, 95% CI: 1.00–1.02). This suggests that individuals who previously sustained longer periods of abstinence may have retained behavioral and motivational resources that facilitate subsequent success [54]. Although the effect size is small and the predictive value of prior abstinence duration has been inconsistent across studies, Borland et al. provide empirical support for this positive association [54].

Notably, while the unadjusted between-group comparison for treatment efficacy did not reach significance (Table 2, *p* = 0.20), the covariate-adjusted model identified a significant advantage for bupropion plus NRT over cytisine. This discrepancy is not unusual; adjusting for baseline covariates (Table 1) enhances statistical precision and can reveal specific pairwise differences that unadjusted multi-group comparisons may lack the power to detect.

Conversely, smoking intensity (cigarettes per day or pack-years) did not emerge as a significant predictor in our adjusted model. In other cohorts, lighter smokers or those with lower cumulative exposure have been found to achieve higher success rates [56,57]. The absence of this association in our data likely reflects the relatively narrow and high range of daily consumption in our sample (mean approximately 26–28 cigarettes/day across groups), a potential ceiling effect, or insufficient power once correlated variables were included in the multivariable model. It should not be interpreted as evidence that smoking intensity is broadly unimportant to cessation outcomes. Indeed, in a Turkish adolescent cohort, the FTND score was the sole independent predictor of one-month cessation (aOR = 0.807) [58], underscoring that the relevance of nicotine dependence as a predictor may vary considerably by age group and clinical setting.

Finally, although meta-analyses support the superiority of varenicline and the added benefit of combination therapies [40,41,45], these options were unavailable in our context (varenicline not marketed in Turkey since 2021). Accordingly, our findings reflect the realities of resource-constrained settings, where treatment choice is shaped by policy and medication availability at least as much as by clinical evidence.

### 4.3. Generalizability

The findings are likely generalizable to similar middle-income countries where varenicline is unavailable, cytisine is subsidized, and cessation services are delivered through specialized clinics. However, caution is warranted in extrapolating results to populations with different sociocultural norms, healthcare structures, or access to behavioral support. The relatively high education level and employment rate in our sample may also limit applicability to more socioeconomically disadvantaged groups. Beyond the sociodemographic and clinical predictors examined here, interindividual variability in the pharmacokinetics and pharmacodynamics of nicotine and smoking-cessation pharmacotherapies—for example, genetic polymorphisms in nicotine-metabolizing enzymes such as CYP2A6 and CYP2B6, and in nicotinic receptor and dopaminergic pathway genes—has been shown to modulate treatment response and could help explain some of the unexplained variance in our model [59]. A personalized-medicine approach incorporating such pharmacogenomic markers, alongside the patient-level factors identified in this study, represents a promising direction for future research and could ultimately support more individualized selection of smoking-cessation pharmacotherapy in place of the largely uniform prescribing observed in current real-world practice.

## 5. Conclusions

In this real-world, non-randomized prospective cohort of adult smokers in Turkey, patient-level factors—including education level, history of previous quit attempts, and duration of prior abstinence—were identified as independent predictors of self-reported (though biochemically unverified) smoking cessation at 3 months. Bupropion plus NRT combination therapy was also independently associated with significantly higher odds of cessation than cytisine, although this estimate warrants cautious interpretation given the small combination-therapy subgroup and the exploratory nature of the pairwise comparison.

It is important to emphasize, however, that these conclusions are specific to short-term outcomes; long-term abstinence was not assessed in this study, and therefore no inference can be drawn regarding sustained cessation beyond the 3-month follow-up.

Notwithstanding this limitation, our findings support the prioritization of individualized, behaviorally informed cessation strategies alongside pharmacotherapy—particularly in resource-constrained settings where first-line agents such as varenicline are not readily accessible. At the same time, they underscore the urgent need for future randomized controlled trials or propensity-score-matched studies with extended follow-up, biochemical confirmation of abstinence, and more comprehensive adjustment for potential confounders, in order to confirm the direction and robustness of the associations reported herein.

Taken together, these data advocate for an integrated, multidisciplinary smoking-cessation model that combines pharmacotherapy with structured behavioral counseling and, when clinically indicated, psychosocial support, rather than focusing exclusively on medication selection. Such an approach is more congruent with current clinical practice and multidisciplinary treatment guidelines, and is likely to yield more meaningful real-world cessation outcomes than the isolated optimization of pharmacotherapeutic choice alone.

## Figures and Tables

**Table 1:** Sociodemographic characteristics of the participants.

Characteristic	Cytisine (*n* = 243)	Bupropion (*n* = 130)	NRT (*n* = 8)	Bupropion + NRT (*n* = 38)	Non-Pharmacological (*n* = 68)	Total (*n* = 487)
Age, years	37.2 ± 9.9	43.1 ± 12.9	42.5 (33–63)	43.0 (18–73)	35.5 (20–68)	39.0 (18–74)
Female	39.9 (97)	45.4 (59)	25.0 (2)	65.8 (25)	52.9 (36)	45.0 (219)
Male	60.1 (146)	54.6 (71)	75.0 (6)	34.2 (13)	47.1 (32)	55.0 (268)
Single	42.4 (103)	37.7 (49)	25.0 (2)	42.1 (16)	42.6 (29)	40.9 (199)
Married	57.6 (140)	62.3 (81)	75.0 (6)	57.9 (22)	57.4 (39)	59.1 (288)
Illiterate	0.8 (2)	2.3 (3)	0.0 (0)	2.6 (1)	0.0 (0)	1.2 (6)
Primary school	17.7 (43)	33.8 (44)	25.0 (2)	23.7 (9)	16.2 (11)	22.4 (109)
High school	28.4 (69)	23.8 (31)	25.0 (2)	42.1 (16)	36.8 (25)	29.4 (143)
University	53.1 (129)	40.0 (52)	50.0 (4)	31.6 (12)	47.1 (32)	47.0 (229)
Currently employed	75.3 (183)	55.4 (72)	75.0 (6)	44.7 (17)	63.2 (43)	65.9 (321)
Currently not employed	24.7 (60)	44.6 (58)	25.0 (2)	55.3 (21)	36.8 (25)	34.1 (166)

NRT = nicotine replacement therapy. Values are presented as means ± SDs, medians (min–max), or percentages (*n*). Row groups: Age; Gender (Female/Male); Marital status (Single/Married); Educational status (Illiterate/Primary school/High school/University); Occupational status (Currently employed/not employed).

**Table 2:** Smoking behaviors and cessation outcomes of the participants by treatment type.

Characteristic	Cytisine	Bupropion	NRT	Bupropion + NRT	Non-Pharmacological	Total	*p*
Duration of smoking, years	17.0 (1–47)	22.5 (1–50)	18.5 (3–33)	22.0 (3–45)	19.0 (3–50)	20.0 (1–50)	0.11
Age at smoking initiation, years	18.3 ± 4.5	18.5 ± 4.9	18.0 ± 4.1	18.3 ± 5.0	18.3 ± 4.8	18.3 ± 4.7	0.99
Packs smoked per day	1.3 ± 0.5	1.2 ± 0.4	1.3 ± 0.4	1.2 ± 0.5	1.3 ± 0.5	1.2 ± 0.5	0.34
Previous quit attempt—Yes	73.3 (178)	77.7 (101)	100.0 (8)	78.9 (30)	67.6 (46)	74.5 (363)	0.17
Previous quit attempt—No	26.7 (65)	22.3 (29)	0.0 (0)	21.1 (8)	32.4 (22)	25.5 (124)	
Duration of quitting, months	1.0 (0–240)	1.0 (0–120)	2.5 (0–24)	3.5 (0–120)	1.0 (0–36)	1.0 (0–240)	0.14
Previous cessation treatment—Yes	32.5 (79)	42.3 (55)	25.0 (2)	31.6 (12)	29.4 (20)	34.5 (168)	0.27
Previous cessation treatment—No	67.5 (164)	57.7 (75)	75.0 (6)	68.4 (26)	70.6 (48)	65.5 (319)	
FTND score	6.28 ± 1.7	6.43 ± 2.0	7.38 ± 1.1	6.05 ± 2.1	6.24 ± 1.9	6.31 ± 1.8	0.36
Cessation status—Quit	32.4 (69)	26.9 (35)	25.0 (2)	47.4 (18)	35.0 (14)	32.2 (138)	0.20
Cessation status—Did not quit	67.6 (144)	73.1 (95)	75.0 (6)	52.6 (20)	65.0 (26)	67.8 (291)	

NRT = nicotine replacement therapy; FTND = Fagerström Test for Nicotine Dependence. Values are presented as means ± SDs, medians (min–max), or percentages (*n*). *p*-values are based on ANOVA, chi-square, or Kruskal–Wallis tests as appropriate; significance level *p* < 0.05.

**Table 3:** Multivariable logistic regression analysis for factors associated with smoking cessation at the third month (treatment type entered as indicator variables; reference category = Cytisine).

Variable	B	SE	Wald	*p*	OR	95% CI (Lower–Upper)
Cytisine	—	—	—	—	Reference	—
Bupropion	−0.092	0.267	0.117	0.732	0.913	0.541–1.540
NRT	−0.369	0.849	0.189	0.664	0.691	0.131–3.651
Bupropion + NRT	0.859 *	0.391	4.813	0.028	2.360	1.096–5.083
Non-pharmacological	0.261	0.375	0.484	0.487	1.298	0.623–2.705
Age	−0.001	0.011	0.002	0.960	0.999	0.978–1.021
Sex	0.128	0.240	0.282	0.595	1.136	0.709–1.820
Education level	0.319 *	0.151	4.447	0.035	1.376	1.023–1.851
Employment status	−0.030	0.270	0.013	0.911	0.970	0.572–1.646
Previous quit attempt	0.759 *	0.320	5.612	0.018	2.137	1.140–4.004
Number of quit attempts	−0.138	0.089	2.429	0.119	0.871	0.732–1.036
Duration of previous abstinence, months	0.009 *	0.004	4.515	0.034	1.009	1.001–1.018
FTND score	0.004	0.058	0.006	0.940	1.004	0.897–1.125
Constant	−2.368 *	0.930	6.481	0.011	0.094	—

* *p* < 0.05. Treatment type was entered as four indicator (dummy) variables, with cytisine as the reference category, rather than as a single linearly coded (0–4) variable. Education level was entered as an ordinal variable (1 = illiterate, 2 = primary school, 3 = high school, 4 = university); an OR above 1 here indicates higher odds of cessation with each higher educational category—the opposite direction from the previously reported (misspecified) model. Given the small NRT subgroup (*n* = 8), the NRT estimate is imprecise and should be interpreted cautiously. Model summary: −2 Log likelihood = 509.203; model *χ*^2^ = 25.909, df = 12, *p* = 0.011; Cox and Snell *R*^2^ = 0.059; Nagelkerke *R*^2^ = 0.083. Classification accuracy: overall 68.8% (sensitivity 10.2%, specificity 96.5%). Hosmer–Lemeshow test: *χ*^2^ = 4.086, df = 8, *p* = 0.849. VIF ranged from 1.02 to 1.43 across predictors.

## Data Availability

The data presented in this study are available on request from the corresponding author due to privacy and ethical restrictions on patient-level clinical data.
